# Predictors of post-stroke delirium incidence and duration: Results of a prospective observational study using high-frequency delirium screening

**DOI:** 10.1177/17474930221109353

**Published:** 2022-07-21

**Authors:** Robert Fleischmann, Tina Andrasch, Sina Warwas, Rhina Kunz, Stefan Gross, Carl Witt, Johanna Ruhnau, Antje Vogelgesang, Lena Ulm, Annerose Mengel, Bettina von Sarnowski

**Affiliations:** 1 Department of Neurology, University Medicine Greifswald, Greifswald, Germany; 2 Friedrich Loeffler Institute of Medical Microbiology, University Medicine Greifswald, Greifswald, Germany; 3 Department of Neurology and Stroke, Hertie-Institute for Clinical Brain Research, Eberhard-Karls University of Tübingen, Tübingen, Germany; 4 Department of Internal Medicine B, University Medicine Greifswald, German Centre for Cardiovascular Research (DZHK), partner site Greifswald, Germany

**Keywords:** Stroke, delirium, risk factors, duration, incidence

## Abstract

**Background:**

Post-stroke delirium (PSD) is a modifiable predictor for worse outcome in stroke. Knowledge of its risk factors would facilitate clinical management of affected patients, but recently updated national guidelines consider available evidence insufficient.

**Aims:**

The study aimed to establish risk factors for PSD incidence and duration using high-frequency screening.

**Methods:**

We prospectively investigated patients with ischemic stroke admitted within 24 h. Patients were screened twice daily for the presence of PSD throughout the treatment period. Sociodemographic, treatment-related, and neuroimaging characteristics were evaluated as predictors of either PSD incidence (odds ratios (OR)) or duration (PSD days/unit of the predictor, *b*), using logistic and linear regression models, respectively.

**Results:**

PSD occurred in 55/141 patients (age = 73.8 ± 10.4 years, 61 female, National Institutes of Health Stroke Scale (NIHSS) = 6.4 ± 6.5). Age (odds ratio (OR) = 1.06 (95% confidence interval (CI): 1.02–1.10), *b* = 0.08 (95% CI = 0.04–0.13)), and male gender (*b* = 0.99 (95% CI = 0.05–1.93)) were significant non-modifiable risk factors. In a multivariable model adjusted for age and gender, presence of pain (OR < sub > *mvar* </sub >= 1.75 (95% CI = 1.12–2.74)), urinary catheter (OR < sub > *mvar* </sub > = 3.16 (95% CI = 1.10–9.14)) and post-stroke infection (PSI; OR < sub > *mvar* </sub > = 4.43 (95% CI = 1.09–18.01)) were predictors of PSD incidence. PSD duration was impacted by presence of pain (*b* < sub > *mvar* </sub >= 0.49 (95% CI = 0.19–0.81)), urinary catheter (*b* < sub > *mvar* </sub > = 1.03 (95% CI = 0.01–2.07)), intravenous line (*b* < sub > *mvar* </sub >= 0.36 (95% CI = 0.16–0.57)), and PSI (*b* < sub > *mvar* </sub >= 1.60 (95% CI = 0.42–2.78)). PSD (OR = 3.53 (95% CI = 1.48–5.57)) and PSI (OR = 5.29 (95% CI = 2.92–7.66)) independently predicted inferior NIHSS at discharge. Insular and basal ganglia lesions increased the PSD risk about four- to eight-fold.

**Discussion/Conclusion:**

This study identified modifiable risk factors, the management of which might reduce the negative impact PSD has on outcome.

## Introduction

Ischemic stroke is becoming increasingly prevalent with an aging population; yet even most advanced reperfusion therapies are viable in only about 20% of patients.^1,2^ There is hence a pivotal role for the management of secondary complications to enhance long-term outcome in patients not amenable for or with unsuccessful reperfusion attempts. Post-stroke delirium (PSD) affects up to 39% of patients with ischemic stroke, is associated with inferior functional and cognitive outcome, and poses a critical, yet modifiable, predictor for poor recovery.^3,4^ Unfortunately, PSD management is generally unstandardized, affected patients remain unrecognized, and initiation of pharmacological and non-pharmacological interventions is delayed.^3,5^ While it remains to be clarified to what extent interventional approaches can reduce the impact of manifest PSD, prevention of PSD is effective and can avert up to one-third of cases.^4,6^ Prevention could be facilitated by knowledge of risk factors for PSD since patients at risk could be easier identified and effects of modifiable risk factors mitigated.

We performed a review of studies that investigated risk factors for PSD (Supplemental Table 1), which generally identified age, stroke severity, infection, deliriogenic medication, and pre-stroke cognitive or functional impairment. Unfortunately, most studies are methodologically biased (retrospective designs, inappropriate choice of screening tools or frequency) and none evaluated risk factors for PSD duration, which substantially impacts neurocognitive outcome following delirium.^7^ Consequently, recently updated German guidelines on acute stroke management concluded that evidence for the clinical management of PSD is insufficient.^8^

## Aims and hypotheses

This study made use of a pre-defined set of endpoints gathered during the investigation of the optimal screening approach for PSD, which we already published elsewhere.^3^ We built upon the meticulous high-frequency PSD detection efforts to establish, importantly modifiable treatment-related, risk factors for PSD incidence and duration in patients with transient ischemic attack (TIA) and ischemic stroke. We hypothesized that risk factors for other types of delirium would also apply in PSD; alternatively, the direct (ischemic) brain injury might outmatch their impact.

## Materials and methods

### Study registration and data availability

This observational trial was prospectively registered at clinicaltrials.gov (NCT03930719), approved by the Institutional Ethics Review Board of the University Medicine Greifswald (BB 031/19) and adhered to the standards of the Helsinki Declaration in its latest revision. Data that support the findings of this study are available from the corresponding author upon reasonable request.

### Setting and study population

The study was conducted on a dedicated Acute Stroke Unit for a continuous 3-month period starting in May 2019, including weekends and public holidays. Patients presenting with ischemic stroke or high-risk TIA defined by an ABCD2 score of ⩾6 within 24 h were eligible for enrollment. There were no further inclusion or exclusion criteria. Data collection was allowed to start immediately on admission to avoid a selection bias. Nonetheless, patients, or their legal representative, had to provide written informed consent to allow use of collected data for study purposes.

### Study design and delirium assessment

The presence of PSD was evaluated in the morning and late shift by two board certified neurologists using the *Diagnostic and Statistical Manual of Mental Disorders* (5th ed.; DSM-5) criteria, and complemented by chart reviews to assess night shifts, throughout the in-hospital treatment period. PSD incidence was defined as the rate of patients rated PSD positive at least once. PSD duration was defined as the number of days patients were at least once rated delirious.

### Predictors of delirium incidence and duration

The patients’ sociodemographic characteristics (age, gender, education, family and working status, education, living situation, religion), substance use, and medical history were scaled as recommended by the National Institutes of Neurological Disorders and Stroke common data elements initiative.^9^ Pain was assessed using the Critical Care Pain Observation Tool (CPOT).^10^ Mobility was assessed using the 10-score intensive care unit (ICU) mobility scale.^11^ Treatment-related risk factors furthermore included presence of nasogastral tubes, urinary catheters, and intravenous lines. Post-stroke infection (PSI) was considered present if it was documented by the treating physician in the daily progress report, and appropriate action was taken (e.g. diagnostic workup, antibiotics). Characteristics of stroke such as National Institutes of Health Stroke Scale (NIHSS) were not evaluated in detail, but used as covariates when applicable, since their impact on PSD is well-established.^3^ Stroke etiologies were defined using *Trial of Org 10172 in Acute Stroke Treatment* criteria. We finally assessed whether PSD, infection, or stroke severity were main predictors of neurological deficits at discharge.

### Data management, sample size considerations, and statistics

Statistical evaluations of non-imaging data were done using SPSS (Version 25, IBM, Armonk, NY, USA). Descriptive statistics are reported as mean values and standard deviations. Binary data are reported as frequencies. Data distribution was confirmed using histograms. The results from inferential statistics are reported with their appropriate coefficients and, if applicable, odds ratios (ORs) including 95% confidence intervals (CIs) in brackets and along with *p*-values denoting the statistical significance. All tests were two-tailed since neither direction of effects should be excluded a priori.

Logistic regression was used to evaluate whether risk factors were significant predictors of PSD incidence (binary outcome). Linear regression was used to test the hypothesis that risk factors are predictors of PSD duration (continuous outcome). We additionally conducted multivariable stepwise backward conditional (threshold: *p* = 0.157)^12^ regression analyses including all significant sociodemographic and treatment-related univariable predictors, adjusted for age, and gender, to estimate their independent predictive value (coefficient subscript: *mvar*). The Akaike information criterion (AIC) was used to assess over- and under-fitting of multivariable models.

### Neuroimaging analysis and statistics

In patients, who received magnetic resonance imaging (MRI) as part of their diagnostic workup, exploratory analyses of lesion locations and volumes were performed. Lesion acuity was confirmed using diffusion-weighted images. Lesion extent was marked-up as region-of-interest (ROI) in corresponding T2-weighted images using MRIcron (v1.0.20190902, University of South Carolina, USA). ROI volumes and locations were calculated in common space using MATLAB (R2018a, MathWorks, Natick, MA, USA). Anatomical locations were statistically compared between delirious and non-delirious patients regarding PSD incidence (Fisher’s exact test) and duration (Wilcoxon’s rank sum test).

## Results

We enrolled 141 patients (73.8 ± 10.4 years of age, 61 female) with an NIHSS score of 6.4 ± 6.5 and modified Rankin Scale (mRS) score of 1.6 ± 1.2 on admission. The study population represents about 75% of ischemic stroke and 12% of TIA patients admitted during this period (mean age = 74 years, mean NIHSS = 7.0). Patients were affected by anterior circulation (*n* = 89), posterior circulation (*n* = 25), lacunar strokes (*n* = 20), and high-risk TIAs (*n* = 7). Stroke etiologies were cardioembolism (*n* = 52), large artery atherosclerosis (*n* = 24), small-vessel occlusion (*n* = 23), and others (*n* = 11; undetermined: *n* = 31). PSD occurred in 39% of patients (55/141).

### Sociodemographic risk factors for PSD incidence and duration

The results of unadjusted univariable and adjusted multivariable analyses of sociodemographic risk factors are summarized in Table 1. Multivariable stepwise regression analyses did not reveal any sociodemographic risk factor other than age and gender for PSD incidence and duration. Education duration was a significant univariable but not multivariable predictor, which is possibly explained by a significant inverse correlation of age and education duration (*ρ* = −0.28, *p* *<* 0.01).

**Table 1.** Sociodemographic risk factors for PSD incidence and duration.

Risk factor categories (risk factors)	Incidence_unadjusted_ Odds ratio (95% CI)	*p*-value	Incidence_multivariable_ Odds ratio (95% CI)	*p*-value	Duration_unadjusted_ Beta coefficient (95% CI)	*p*-value	Duration_multivariable_ Beta coefficient (95% CI)	*p*-value	Interpretation
Demographic risk factors									
Age	***1.06 (1.02 to 1.10)***	***<0.01***	***1.05 (1.01 to 1.09)***	***0.02***	***0.08 (0.03 to 0.12)***	***<0.01***	***0.08 (0.04 to 0.13)***	***<0.01***	***PSD risk duration increases with age***
Gender	0.67 (0.32 to 1.39)	0.28	0.44 (0.19 to 1.03)	0.06	**−*0.78 (−1.61 to 0.01)***	***0.05***	**−*0.99 (*−*1.93 to −0.05)***	***0.04***	***PSD shorter in females***
Socioeconomic risk factors									
Education type	−0.36 (−2.51 to 1.79)	0.74	. . .	. . .	−0.36 (−2.51 to 1.78)	0.96	. . .	. . .	No risk factor for PSD
Education duration	***0.81 (0.65 to 1.00)***	***0.05***	0.86 (0.69 to 1.08)	0.20	−0.17 (−0.41 to 0.08)	0.18	. . .	. . .	Not an independent risk factor for PSD
Work status	0.29 (−2.43 to 3.03)	0.83	. . .	. . .	0.29 (−2.43 to 3.03)	0.73	. . .	. . .	No risk factor for PSD
Family status	−1.58 (−5.2 to 2.02)	0.74	. . .	. . .	−1.58 (−5.16 to 2.09)	0.59	. . .	. . .	No risk factor for PSD
Living situation	−0.59 (−2.68 to 1.51)	0.58	. . .	. . .	−0.59 (−2.68 to 1.51)	0.43	. . .	. . .	No risk factor for PSD
Religion (any)	−0.28 (−1.36 to 0.80)	0.62	. . .	. . .	−0.28 (−1.36 to 0.80)	0.76	. . .	. . .	No risk factor for PSD
Substance use risk factors									
Alcohol (amount/month)	1.51 (0.61 to 3.78)	0.38	. . .	. . .	−0.13 (−1.74 to 1.47)	0.87	. . .	. . .	No risk factor for PSD
Smoking (indicator)	1.71 (0.59 to 4.98)	0.54	. . .	. . .	−0.46 (−1.79 to 0.86)	0.49	. . .	. . .	No risk factor for PSD

PSD: post-stroke delirium; 95% CI: 95% confidence interval.

… denotes not included in the multivariable model.

Environmental factors are known to be important predisposing and, except for substance use, non-modifiable factors contributing to the development of delirium, but their impact on PSD remained unclear. We identified age as a risk factor for developing an episode of PSD that is also longer. Female gender is associated with shorter duration. The multivariable model included all significant univariable factors, adjusted for age, and gender, and revealed that education duration was not an independent risk factor. Significant factors for either PSD incidence or duration are highlighted in bold and italic font.

### Treatment-related risk factors for PSD incidence and duration

Results of unadjusted univariable and adjusted multivariable analyses of treatment-related risk factors are summarized in Table 2. The multivariable model including significant univariable predictors, and additionally adjusted for age and gender, revealed that presence of an indwelling urinary catheter, pain, and PSI were independent predictors of an episode of PSD. The presence of an intravenous line only affected PSD duration but not its incidence. The inclusion of stroke severity did, based on the AIC, not improve the logistic or linear regression model, nor did it change the significance of included predictors for PSD incidence and duration.

**Table 2.** Impact of treatment-related parameters on PSD incidence and duration.

Risk factor categories (risk factors)	Incidence_unadjusted_ Odds ratio (95% CI)	*p*-value	Incidence_multivariable_ Odds ratio (95% CI)	*p*-value	Duration_unadjusted_ Beta coefficient (95% CI)	*p*-value	Duration_multivariable_ Beta coefficient (95% CI)	*p*-value	Interpretation
Treatment-related devices									
Nasogastral tube (indicator)	1.74 (0.27 to 11.36)	0.56	. . .	. . .	0.43 (−1.13 to 1.98)	0.59	. . .	. . .	No risk factor for PSD
Intravenous line (indicator)	1.02 (0.82 to 1.27)	0.85	. . .	. . .	***0.35 (0.15 to 0.56)***	***<0.01***	***0.36 (0.16 to 0.57)***	***<0.01***	***PSD longer if present***
Urine catheter (indicator)	***6.13 (2.52 to 14.91)***	***<0.01***	***3.16 (1.10 to 9.14)***	***0.03***	***1.75 (0.82 to 2.67)***	***<0.01***	***1.03 (0.01 to 2.07)***	***0.05***	***PSD risk/duration increases if present***
Environmental factors									
Pain	***1.86 (1.28 to 2.70)***	***<0.01***	***1.75 (1.12 to 2.74)***	***0.02***	***0.54 (0.22 to 0.87)***	***<0.01***	***0.49 (0.19 to 0.81)***	***<0.01***	***PSD risk/duration increases with pain***
Mobility^a^	1.10 (−0.71 to 2.28)	0.07	. . .	. . .	−0.52 (−2.38 to 1.33)	0.58	. . .	. . .	No risk factor for PSD
Infection (indicator)	***4.90 (2.14 to 11.23)***	***<0.01***	***4.43 (1.09 to 18.01)***	***0.04***	***2.89 (1.84 to 3.93)***	***<0.01***	***1.60 (0.42 to 2.78)***	***0.01***	***PSD risk/duration increases with infection***

PSD: post-stroke delirium; 95% CI: 95% confidence interval.

… denotes not included in the multivariable model.

The treatment environment and associated medical procedures are established risk factors for delirium but not PSD. We found that presence of a urine catheter, more severe pain, and post-stroke infection not only increased the odds for an episode of PSD, but were also, in addition to the presence of an intravenous line, associated with longer duration of PSD. Significant risk factors for either PSD incidence or duration are highlighted in bold and italic font. Significant univariable factors were included in a multivariable model, which was also adjusted for age and gender, to assess their independent impact on PSD.

a Corrected for stroke severity.

C-reactive protein (CRP) levels were associated with higher odds for an episode of PSD (OR = 1.02 (95% CI = 1.00 to 1.05), *p* = 0.04) and longer duration (*b* = 0.02 (95% CI = 0.00 to 0.03), *p* = 0.02). Analyses of variance yielded a significant association of CRP and both the presence of PSD (*F*(1, 140) = 4.9, *p* = 0.03) and PSI (*F*(1, 140) = 49.5, *p* < 0.01). Routine cell counts neither predicted PSD incidence nor duration.

Given that PSD and PSI interact, and both are established to affect recovery, we were interested whether PSD and PSI independently predicted NIHSS at discharge. We found that both independently predicted worse NIHSS at discharge in a multivariable model adjusted for age, gender, and NIHSS on admission (PSD: OR_*mvar*_ = 3.53 (95% CI = 1.48–5.57), *p_mvar_* < 0.01; PSI: OR_*mvar*_ = 5.29 (95% CI = 2.92–7.66), *p_mvar_* < 0.01).

### Neuroimaging

There were 44/141 patients, who received an MRI as part of clinical routine. Lesion volume was larger in patients with PSD (5.07 mL (95% CI = 1.01 to 11.58), *p* = 0.05) and associated with PSD duration (*b* = 0.15 (95% CI = 0.08 to 0.21), *p* *<* 0.01). The results from exploratory lesion mapping are summarized in Supplemental Figure 1 and Supplemental Table 2, and indicate that insular and basal ganglia lesions might increase the risk for PSD.

## Discussion

The study population represents approximately 75% of patients admitted with ischemic stroke during the study period and was furthermore representative regarding age and stroke severity. Our findings enhance the class of evidence that modifiable and non-modifiable risk factors for delirium of other causes also apply for PSD incidence, including age, male gender, and treatment-related factors such as pain, medical devices, and infection. We furthermore yielded novel evidence that most risk factors also affect PSD duration. Exploratory imaging analyses revealed that insular and basal ganglia lesions may increase the risk for PSD. In a multivariable analysis, PSD and PSI independently predicted worse outcome at discharge.

### Comparison of identified risk factors with previous studies and practical implications

Non-modifiable risk factors were similar to what would be expected in general delirium, that is, the risk increases in males and older patients.^13^ Previous studies with state-of-the-art diagnostic approaches to PSD found that older age but not gender was a significant risk factor. Yet, a study that investigated a multicomponent delirium prevention intervention following stroke also found that males were more often affected, which confirms our finding.^14^ A previous study furthermore identified lower education was a univariable risk factor in PSD, yet neither the previous nor our study confirmed this in the multivariable analysis.^15^ In our sample, this redundancy of the predictor was explained by the significant correlation of age and lower education.

None of the previous studies investigated the influence of treatment-related patterns including pain and presence of medical devices on the occurrence of PSD. This is somewhat surprising, since ICU delirium is known to be critically affected by pain and noxious stimuli induced by the application of medical devices including urinary catheter and intravenous lines.^16^ Our study confirmed that these are also relevant for PSD. Hence, medical devices should not be used longer than necessary, and patients should be carefully assessed for pain.

Substance use neither influenced the incidence nor the duration of PSD. Previous studies often excluded patients with known alcohol misuse leaving only a few studies that investigated the influence of alcohol on PSD (see Supplemental Table 1) and only one study reported that the combination of prior drug and/or alcohol misuse increased the odds for PSD about 2.5-fold.^17^ It remains elusive how much of this effect was due to alcohol, which renders comparisons with our data difficult, but alcohol (withdrawal) certainly is an acknowledged risk factor for any type of delirium.

### Implications of neuroimaging for research and practice

Every patient with presumed ischemic stroke is assessed with neuroimaging rendering it readily available to assess the risk for PSD.^18^ Exploratory analyses revealed that larger lesion volume and insular or basal ganglia lesion locations might increase the risk for PSD. Clinical utility of these findings needs to be prospectively validated, importantly since CT is often used instead of MRI.

### Interaction of PSD and PSI

It is well-established that PSD and PSI are associated with inferior outcome following ischemic stroke.^18,19^ We found that PSD and PSI furthermore independently predict inferior outcome at discharge after correction for stroke severity on admission, which deserves close attention. While delirium and infection are known to interact, importantly one being a risk factor for the other, their individual or mutual impact on restitution following stroke remains to be elucidated, and thus both entities should be closely monitored, and potentially adjusted for, in interventional clinical trials that aim to improve neurological outcome by influencing either PSI or PSD.

### Limitations

The investigation of risk factors was not the primary endpoint of this study, and hence, it was not powered to test these as dedicated primary hypothesis. Nonetheless, all evaluated risk factors were pre-defined secondary endpoints in the study registration, and there were no post hoc revisions of secondary endpoints, which should minimize the error. While we may have missed risk factors with effect sizes lower than Cohen’s *d* of 0.48 (sensitivity based on an alpha of 0.05 and power of 0.80), we yielded modifiable risk factors that are in line with risk factors in ICU delirium and single PSD studies. Another concern might be that our definition of PSI was not pre-specified. Consequently, some PSD cases may have been caused by minor infections rendering the independent contribution of PSD and PSI on inferior outcome lower than found in this study.^20^ While we believe that this effect should be minor, PSI needs to be more meticulously defined in future studies. Furthermore, the severity of systemic inflammatory response (SIRS) may impact on PSD with or without overt infection,^20^ and should thus be monitored using established scores, for example, the National Early Warning Score.

## Conclusion

We confirmed that non-modifiable and modifiable risk factor established for delirium in patients not affected by stroke also hold true for PSD. The identification of modifiable risk factors—importantly pain and presence of medical devices—has immediate practical implications and should be included in the clinical management of stroke patients. Particular neuroimaging findings might increase the awareness for an episode of PSD. In future studies, PSD and PSI should both be meticulously investigated since both are common complications of ischemic stroke and independently predict inferior outcome. Neglecting one or the other might negatively impact the interpretation of interventional trials if they are not controlled for.

## Supplemental Material

sj-pdf-1-wso-10.1177_17474930221109353 – Supplemental material for Predictors of post-stroke delirium incidence and duration: Results of a prospective observational study using high-frequency delirium screening Click here for additional data file. Supplemental material, sj-pdf-1-wso-10.1177_17474930221109353 for Predictors of post-stroke delirium incidence and duration: Results of a prospective observational study using high-frequency delirium screening by Robert Fleischmann, Tina Andrasch, Sina Warwas, Rhina Kunz, Stefan Gross, Carl Witt, Johanna Ruhnau, Antje Vogelgesang, Lena Ulm, Annerose Mengel and Bettina von Sarnowski in International Journal of Stroke
