# Supplementary material for: Predictors of post-stroke delirium incidence and duration: Results of a prospective observational study using high-frequency delirium screening
Source: Int J Stroke. 2022 Jul 21;18(3):278–84. doi: 10.1177/17474930221109353 (PMC9940154; doi:10.1177/17474930221109353)
Supplement: sj-pdf-1-wso-10.1177_17474930221109353 – Supplemental material for Predictors of post-stroke delirium incidence and duration: Results of a prospective observational study using high-frequency delirium screening [file sj-pdf-1-wso-10.1177_17474930221109353.pdf]

Supplemental table 1

Screenings once daily: **5**	Screenings with DSM 3 or 4: **9**	Studies with severe selections bias (e.g. exclusion of dementia or aphasia): **15**
Screenings > once daily: **5**	Screenings with DSM 5: **6**	Studies that stated aphasia as associated with delirium: **2**
Screenings perfomed by ward staff, included in routine work (> once daily): **4**	Screenings with DSM confirmation: **1**	At least daily screenings using DSM 5 for diagnosis delirium: **6**

REFERENCE	STUDY	N	PSD INCIDENCE %	SCREENING FREQUENCY	SCREENING METHOD	PROSPEKTIVE Y - YES N - NO	INDEPENDENT VARIABLES	EXCLUSION CRITERIA	INCLUDEDLOW RISK TIA Y - YES N - NO / - NOT MENTIONED	NOTES
28320171<br>Ojagbemi et al.,<br>J Neurol Sci.,<br>2017<br>[1]	Stroke severity predicts postStroke Delirium and its association with Dementia: Longitudinal observation from a low income setting	101	33	2 in 1st week	- CAM<br>- DRS<br>- MMSE<br>- memory tests<br>- ADL	y	- severe Stroke	- Aphasia<br>- severe comorbidities	/	- Stroke Levity Scale does not investigate sensory deficits
31554501<br>Shaw et al.,<br>Stroke.,<br>2019<br>[2]	Occurrence Rate of Delirium in Acute Stroke Settings: Systematic Review and Meta-Analysis	6718<br>32 paper	- 25 in summary<br>- 23 (studies at low risk bias)<br>- 21 (using CAM)<br>- 27 (using DSM)<br>- 32 (other assessment strategies)<br><br>- 6,7-61%	1st day possibly repeated until day 5	- CAM<br>- DSM<br>- others	y/n	-	- Dementia<br>- severe comorbidities<br>- Aphasia<br>- < 20 patients	y	- recommend one time point assessment
31554500<br>Shaw et al.,<br>Stroke.,<br>2019<br>[3]	Delirium in an Acute Stroke Setting, Occurence, and Risk Factors	708	26,4	1 within first 48h, possibly repeated if necessary until day 5	- DSM 5<br>- 4AT<br>- clinical investigation<br>- Hopkinson Abbreviated Mental Test<br>- GP-Cog7 informed questionnaire<br>- medical records	y	- Age<br>- NIHSS<br>- Drugs/Alcohol	- SAH	y	- no data for Aphasia
28828209<br>Nydahl et al.,<br>Brain Behav.,<br>2017<br>[4]	Prevalence for Delirium in Stroke patients: A prospective controlled study	309	10,7	3 / day by nurses during routine care	- CAM	y	-	- > 24h admission<br>- < 18years<br>- unable to be assessed<br>- foreign language<br>- previous neuroradiological interventions	y	- screenings by ward nurses during their daily work<br>- CVT included

REFERENCE	STUDY	N	PSD INCIDENCE %	SCREENING FREQUENCY	SCREENING METHOD	PROSPEKTIVE Y - YES N - NO	INDEPENDENT VARIABLES	EXCLUSION CRITERIA	INCLUDEDLOW RISK TIA Y - YES N - NO / - NOT MENTIONED	NOTES
29423616<br>Pasinska et al.,<br>J Neurol.,<br>2018<br>[5]	Frequency and predictors of post Stroke Delirium in PRospective Observational POLIsh Study (PROPOLIS)	750	27,07	1 / day during the first week	- bCAM<br>- CAM_ICU when Aphasia<br>- DRS<br>- DSM5 based criteria<br>- IQCODE<br>- MoCA	y	- MoCA<br>- Neglect<br>- Vision deficit<br>- WBCC<br>- Pre-mRS<br>- Modified Cumulative Illness Rating Scale	- Coma<br>- Brain-tumor<br>- alcoholic withdrawal<br>- CVT<br>- SAH<br>- Trauma<br>- Vasculitis<br>- Malignacy	y	- included admission ≤ 48h of symptom onset<br>- same study population as 26084453
28012839<br>Alvarez-Perez et al.,<br>J Stroke Cerebrovasc Dis.,<br>2016<br>[6]	Prevalence an risk factors for Delirium an acute Stroke patients. A retrospective 5-years clinical series	1161	10,2	-	- DSM 5	n	previous Alzheimers disease	- TIA<br>- CVT	n	
21307355<br>Oldenbeuving et al.,<br>Neurology.,<br>2011<br>[7]	An early prediction of Delirium in the acute phase after Stroke	527	1. 11,8<br>2. 9,7	1. Once at day 2-4<br>2. Once at day 5-7<br>3. continuous until no Delirium detectable	- CAM<br>- DRS	y	simplified model:<br>- NIHSS<br>- right sided lesions<br>- age<br>- infections<br><br>mulivariat:<br>- IQCODE,<br>- right sided lesions<br>- anterior circulation large vessels<br>- NIHSS<br>- infections<br>- cortical atrophy	- TIA<br>- SAH<br>- severe mental retardation<br>- severe language barrier	n	-

REFERENCE	STUDY	N	PSD INCIDENCE %	SCREENING FREQUENCY	SCREENING METHOD	PROSPEKTIVE Y - YES N - NO	INDEPENDENT VARIABLES	EXCLUSION CRITERIA	INCLUDEDLOW RISK TIA Y - YES N - NO / - NOT MENTIONED	NOTES
27999490<br>Dostovic et al.,<br>Mater Sociomed.,<br>2016<br>[8]	Predictors for post-Stroke Delirium Outcome	100 with Delirium	25,3	1. once during the first 24h after admission<br>2. once during the first week<br>3. at discharge time,<br>4. one assessment each after 3, 6 and 12 months<br>5. after recurring Stroke	- DRS<br>- DSM 4<br>- 5 neuropsychological assessments<br>- GCS<br>- NIHSS<br>- MMSE	y	increased mortality for patients >65y and patients with complications like pneumonia, injuries, decubitus, thromboembolia, urinary infections	- epileptic seizure<br>- GCS < 8<br>- Aphasia<br>- early Dementia<br>- alcohol abuse<br>- previous mood disorders<br>- Delirium causing medication<br>- recurrend Stroke<br>- TIA	n	- first assessment and neuropsycholog. assessment at discharge time different rater —> not blinded but no exploration of prevalences<br>- unknown if patients without Delirium developed Delirium between screenings
22672215<br>Miu et al.,<br>Geriater Gerontol Int.,<br>2012<br>[9]	Incidence of post-Stroke Delirium and 1year outcome	314	27,4	1/day for the first 5d	- CAM<br>- DSM3	y	- NIHSS<br>- acute urinary retention<br>- chest infection<br>- prev. cognitive impairment<br>- TACI<br>- POCI	- TIA<br>- CVT<br>- severe head trauma at any time before<br>- GCS < 5<br>- age < 50y<br>- no reliable informant	n	-
30172676<br>Qu et al.,<br>J Stroke Cerebrovasc Dis.,<br>2018<br>[10]	Delirium in the Acute Phase of Ischemic Stroke: Incidence, Risk Factors, and Effects on Functional Outcome	261	14,6	1. testing once at day 1-3<br>2. testing at day 5-7<br>- if CAM was positive, daily DRS-R-98<br>- ADL + mRS after 3 and 6 months	- CAM + —> DRS-R-98<br>- NIHSS<br>- mRS<br>- Lawton ADL	y	- Age<br>- NIHSS<br>- previous Strokes<br>- infections<br>- left cortical infarcts	- TIA<br>- Mental Disorders<br>- hemorrhagic Stroke<br>- CNS-Disorder (Dementia, M. Parkinson)<br>- no MRI investigation possible	n	- Stroke occurring within 7 days before admission<br>- MRI as pre-condition —> selection bias possible

REFERENCE	STUDY	N	PSD INCIDENCE %	SCREENING FREQUENCY	SCREENING METHOD	PROSPEKTIVE Y - YES N - NO	INDEPENDENT VARIABLES	EXCLUSION CRITERIA	INCLUDEDLOW RISK TIA Y - YES N - NO / - NOT MENTIONED	NOTES
30061796 Dostovic et al., Mater Sociomed., 2018 [11]	Mortality and Functional Disability of PostStroke Delirium.	100 with Delirium + matched control group		1. testing once in first week after Stroke onset<br>2. at discharge until first month<br>3. 3 months after Stroke<br>4. 6 months after Stroke<br>5. 12 months after Stroke	- DRS-R-98<br>- DSM 4<br>- GCS<br>- NIHSS<br>- MMSE	y	-	- epileptic seizure<br>- Aphasia<br>- early stage Dementia<br>- alcohol abuse<br>- Mood Disorders<br>- Delirium causing medication<br>- TIA	n	- GCS > 8<br>- mortality and functional impairment investigated
29602617 Hosoya et al., J Stroke Cerebrovasc Dis., 2018 [12]	Association between Delirium and Prehospitalization Medication in PostStroke Patients.	269	36	unknown	- ICDSC ≥ 4	y	- medication classification	- unknown medication history<br>- TIA	n	- screening frequency unknown<br>- included SAH & ICH<br>- included Dementia,<br>- significant for paralysis, Neglect, Aphasia and Somnolence —> stated that Aphasia influences Delirium detection
27816188 Kozak et al., Neurol Neurochir Pol., 2016 [13]	Delirium in patients with acute ischemic Stroke admitted to the non-intensive Stroke unit: Incidence and association between clinical features and inflammatory markers	60	18	1/day by psychiatrist until discharge	DSM 4<br>DRS	y	-	- TIA<br>- ICH<br>- SAH<br>- impaired consciousness<br>- severe Aphasia/Dysphasia<br>- myocardial infarction<br>- renal dysfunction<br>- inflammation last three months<br>- brain tumor<br>- trauma one month<br>- antidepressiv medication/ psychotropics	n	- low incidence according to selection bias possible<br>- significant for length of hospitalization, age, NIHSS, TACI<br>- first 3 days Delirium onset

REFERENCE	STUDY	N	PSD INCIDENCE %	SCREENING FREQUENCY	SCREENING METHOD	PROSPEKTIVE Y - YES N - NO	INDEPENDENT VARIABLES	EXCLUSION CRITERIA	INCLUDEDLOW RISK TIA Y - YES N - NO / - NOT MENTIONED	NOTES
27306854<br>Rice et al.,<br>J Cardiovasc Nurs.,<br>2017<br><br>Cite use of specialized care<br><br>[14]	A Pilot Randomized Controlled Trial of the Feasibility of a Multicomponent Delirium Prevention Intervention Versus Usual Care in Acute Stroke	134	10	- 48h enrollment time slot for inclusion<br>- every 12h (standard of care)<br>- daily CAM by research staff for complication detections	- CAM<br>- MoCA<br>- NIHSS	y	-	- TIA<br>- <50 years<br>- SAH<br>- ICH<br>- barriers in speech<br>- Delirium on admit<br>- Aphasia<br>- medically unstable	n	- significant for male gender and Anticholinergic scales<br>- no incidence detection
23969090<br>Oldenbeuving et al.,<br>Am J Geriater Psychiatry.,<br>2013<br>[15]	Delirium in the Acute Phase after Stroke and the Role of Apolipoprotein E Gene	353	11,8	1. testing once at day 2-4 after admission<br>2. testing once at day 5-7	- CAM<br>- IQCODE	y		- TIA<br>- SAH	n	- Findings: Age, IQCODE
23744891<br>Oldenbeuving et al.,<br>J Neurol Neurosurg Psychiatry.,<br>2013<br>[16]	An early prediction of Delirium in the acute phase after Stroke	273	11,8	1. once at day 2-4 after admission<br>2. once at day 5-7	CAM<br>IQCODE<br>NIHSS	Y		TIA<br>SAH	n	
22571183<br>Kostalova et al.,<br>Brain Inj.,<br>2012<br>[17]	Towards a predictive model for post-Stroke Delirium	100	43	1. testing during the first 24h<br>2. 1/day for 7 days	- DSM 4<br>- CAM-ICU	y	- age<br>- ICH<br>- lesion volume<br>- SOFA score<br>- metabolic abnormalities	- TIA<br>- SAH<br>- neurosurgical interventions<br>- brain tumor<br>- Venous infarction<br>- speech barriers<br>- RASS ≤ -4	n	- Stroke onset ≤24h<br>- Dementia included<br>- age as independant factor from metabolic abnormalities and lesion volume<br>- findings: Dementia, age, number of drugs, AUDIT Score, number of ACH drugs, SOFA, many blood parameters, NIHSS, Lesion volume, TACI, Hemineglect as predictors

REFERENCE	STUDY	N	PSD INCIDENCE %	SCREENING FREQUENCY	SCREENING METHOD	PROSPEKTIVE<br>Y - YES<br>N - NO	INDEPENDENT VARIABLES	EXCLUSION CRITERIA	INCLUDEDLOW RISK TIA<br>Y - YES<br>N - NO<br>/ - NOT MENTIONED	NOTES
22234842<br>Carin-Levy et al.,<br>J Neurol.,<br>2012<br>[18]	Delirium in acute Stroke: screening tools, incidence rates and predictors: a systematic review	20	26	- 10x once within first week<br>- 3x > once<br>- rest not reporting	6x DSM 4, 3xDSM-3R, 2x DSM-4R, 1x DSM3, 3x CAM,<br>14x DRS, DRS-98 , 2x CAM, 2x DRS + CAM, 3x OBS, 2x MMSE, 1x disorientation	both	-	-	-	-
21903955<br>Pendlebury et al.,<br>Stroke.,<br>2011<br>[19]	Transient Cognitive Impairment in TIA and Minor Stroke	280	39	1. testing within 24h after onset<br>2. testing 1 month later	- MMSE	y	-	- recurrent TIA/ Stroke (1 month)<br>- Dementia<br>- speech barriers<br>- Dysphasia<br>- Dysarthria<br>- overt infections<br>- severe hearing or visual impairment that leads to untestability	y	- included patients significantly younger than excluded<br>- Finding: TCI more frequent in acute TIA/Stroke especially with resident focal deficits
26084453<br>Klimiec et al.,<br>BMC Neurol.,<br>2015<br>[20]	PRospective Observational POLIsh Study on post-Stroke Delirium: methodology of hospital-based cohort study on Delirium prevalence, predictors and diagnostic tools	750	k.A.	- 1/day for 7 days after admission<br>- 2 cognitive screenings	- CAM,<br>- DRS-R-98,<br>- Cognitive-Test for Delirium (CTD)<br>- Ward nurses questionnaire according to DSM 5 criteria for diagnosis Delirium<br>- MoCA,<br>- PHQ-9,<br>- mRS,<br>- IQCODE	y	-	- Coma,<br>- brain tumor<br>- alcoholic withdrawal<br>- CVT<br>- SAH<br>- Life expectancy <1year<br>- Vasculitis	y	- included admission ≤ 48h of symptom onset<br>- ward nurses deportations to apply DSM-V criteria<br>- NO results published<br>- selection bias for the elderly due to exclusion of life expectancy < 1 year
33476475<br>Rollo et al.,<br>Eur J Neurol.,<br>2021<br>[21]	Delirium in acute Stroke: A prospective, cross-sectional, cohort-study	120	30	one assessment at admission, that was repeated within 72h or at onset of delirious symptoms	- RASS<br>- CAM-ICU	y	-	- TIA<br>- no lesion in brain imaging<br>- CVT<br>- SAH<br>- ICU-treatment	n	- Inclusion NIHSS ≥1 on admission<br>- Findings: association with Aphasia, deep Fazekas, COPD, physical restraint, diabetes lower incidence of Delirium<br>- no full text version

REFERENCE	STUDY	N	PSD INCIDENCE %	SCREENING FREQUENCY	SCREENING METHOD	PROSPEKTIVE Y - YES N - NO	INDEPENDENT VARIABLES	EXCLUSION CRITERIA	INCLUDEDLOW RISK TIA Y - YES N - NO / - NOT MENTIONED	NOTES
31695347<br>Kotfis et al.,<br>Clin Interv Aging.,<br>2019<br>[22]	Characteristics, risk factors and Outcome of Early-onset Delirium in the elderly patients with first ever acute ischemic Stroke- a prospective observational cohort study	760	16	2/day during hospitalization	- CAM-ICU<br>- DSM-5 criteria for diagnosis	retrospective analysis of a prospective Study	- atrial fibrillation<br>- Hemianopia<br>- high Rankin Score<br>- low PWR	- TIA<br>- previous Stroke<br>- hematologic disorders<br>- incomplete lab data	n	- selection bias by first ever Stroke possible<br>- included admission ≤ 48h of symptom onset
21560162<br>Melkas et al.,<br>Int J Geriater Psychiatry.,<br>2011<br>[23]	Post-Stroke Delirium in relation to Dementia and long-term mortality	263	19	nurses notes, available medical records day 1-7	- DSM 4- Delirium<br>- DSM-3 Dementia<br>- mRS<br>- IADL<br>- Barthel	y	-	- ICH<br>- TIA	n	- no assessments done beside the nursing routine
20586734<br>Dahl et al.,<br>Acta Neurol Scand Suppl.,<br>2010<br>[24]	Delirium in acute Stroke--prevalence and risk factors	178	10	CAM twice daily by trained nurses, if postive DSM-4 by neurologist	- CAM<br>- DSM-4<br>- MDAS —> Serverity<br>- NIHSS<br>- MMSE<br>- mRS	y	- Dementia<br>- Hemianopsia<br>- Apraxia<br>- age<br>- infection	- TIA<br>- terminal stage<br>- unconsciousness<br>- severe Dementia	n	- age, Apraxia and Dementia in multivariate logistic regression<br>- selection bias possible
19388034<br>Mc Manus et al.,<br>Int J Geriater Psychiatry.,<br>2009<br>[25]	The evaluation of Delirium post-Stroke	82	28	- first within 4 days<br>- 1/week up to 4 weeks	- CAM<br>- DRS<br>- MMSE	y	-	speech barriers<br>GCS<8<br>time delay<br>TIA	n	screenings by one physician
17966952<br>Shih et al.,<br>Acta Neurol Taiwan.,<br>2007<br>[26]	Confusion or Delirium in patients with posterior cerebral arterial infarction	29	k.A.	k.A.	k.A.	n	-	k.A.	n	- no full text version<br>- only PCA-infarction included<br>- Finding: confusion and Delirium left PCA bilateral > right PCA<br>- Diabetes associated
16913984<br>Sheng et al.,<br>J Am Geriater Soc.,<br>2006<br>[27]	Delirium within three days of Stroke in a cohort of elderly patients	186	25	Once within first 3 days by geriatricians/ neurologists, information from junior staff, nurses discussed with physicians	- DSM -4	y	-	- TIA<br>- SAH<br>- severe head trauma in history or neurosurgery<br>- tumor<br>- CVT<br>- Coma<br>- Aphasia<br>- advanced Dementia<br>- acute psychotic disorder before Stroke	n	- selection bias<br>- independent factors age and Dementia

REFERENCE	STUDY	N	PSD INCIDENCE %	SCREENING FREQUENCY	SCREENING METHOD	PROSPEKTIVE Y - YES N - NO	INDEPENDENT VARIABLES	EXCLUSION CRITERIA	INCLUDEDLOW RISK TIA Y - YES N - NO / - NOT MENTIONED	NOTES
14991351 Caeiro et al., J Neurol., 2004 [28]	Delirium in the first days of acute Stroke	218	13	trained psychologists on first day	- DRS<br>- DSM-4 confirmation	y	- Neglect<br>- ICH<br>- Age<br>- medical complications	- TIA<br>- GCS < 5	n	- scoring 0 if unable to assess<br>- medical complications and age as independant risk factors
10187878 Hénon et al., Stroke., 1999 [29]	Confusional state in Stroke: relation to preexisting Dementia, patient characteristics, and outcome	202	24	k.a.	- DSM-4<br>- DRS	y	-	- TIA<br>- SAH<br>- CVT<br>- <40y<br>- speech barriers<br>- severe head trauma or neurosurgery in history	n	no full text version available

Abbreviations:

4AT	-	4 A's Test	IADL	-	Instrumental Activities of Daily Living	PHQ	-	Patient Health Questionnaire
ADL	-	Activities of Daily Living	ICDSC	-	Intensiv Care Delirium Screening Checklist	POCI	-	Posterior Circulation Infarction
bCAM	-	brief Confusion Assessment Method	ICH	-	Intracerebral Hemorrhage	PSD	-	Poststroke Delirium
CAM	-	Confusion Assessment Method	ICU	-	Intensiv Care Unit	PWR	-	Platelet-to-White Blood Cell Ratio
CVT	-	Cerebral Venous Thrombosis	IQCODE	-	Informed Questionnaire on Cognitive Decline in the Elderly	RASS	-	Richmond Agitation-Sedation Scale
DSM	-	Diagnostic and Statistical Manual of Mental Disorders	MDAS	-	Memorial Delirium Assessment Scale	SAH	-	Subarachnoid hemorrhage
DRS	-	Delirium Rating Scale	MMSE	-	Minimental State Examination	SOFA	-	Sepsis-related Organ Failure Assessment
DRS-R-98	-	Delirium Rating Scale-revised-98	MoCA	-	Montreal Cognitive Assessment	TACI	-	Total Anterior Circulation Infarction
GCS	-	Glasgow Coma Scale	mRS	-	modified Rankin Scale	TIA	-	Transient Ischemic Attack
GP-COG	-	General Practitioner Assessment of Cognition	NIHSS	-	National Institut of Health Stroke Scale	WBCC	-	White Blood Cell Count

References:

[1] Ojagbemi A, Owolabi M, Bello T, Baiyewu O. Stroke severity predicts poststroke delirium and its association with dementia: Longitudinal observation from a low income setting. J Neurol Sci. 2017 Apr 15;375:376-381. doi: 10.1016/j.jns.2017.02.039. Epub 2017 Feb 20. PMID: 28320171; PMCID: PMC6169523.

[2] Shaw RC, Walker G, Elliott E, Quinn TJ. Occurrence Rate of Delirium in Acute Stroke Settings: Systematic Review and Meta-Analysis. Stroke. 2019 Nov;50(11):3028-3036. doi: 10.1161/STROKEAHA.119.025015. Epub 2019 Sep 26. PMID: 31554501.

[3] Shaw R, Drozdowska B, Taylor-Rowan M, Elliott E, Cuthbertson G, Stott DJ, Quinn TJ. Delirium in an Acute Stroke Setting, Occurrence, and Risk Factors. Stroke. 2019 Nov;50(11):3265-3268. doi: 10.1161/STROKEAHA.119.025993. Epub 2019 Sep 26. PMID: 31554500.

[4] Nydahl P, Bartoszek G, Binder A, Paschen L, Margraf NG, Witt K, Ewers A. Prevalence for delirium in stroke patients: A prospective controlled study. Brain Behav. 2017 Jun 23;7(8):e00748. doi: 10.1002/brb3.748. PMID: 28828209; PMCID: PMC5561309.

[5] Pasinska P, Kowalska K, Klimiec E, Szyper-Maciejowska A, Wilk A, Klimkowicz-Mrowiec A. Frequency and predictors of post-stroke delirium in PRospective Observational POLIsh Study (PROPOLIS). J Neurol. 2018 Apr;265(4):863-870. doi: 10.1007/s00415-018-8782-2. Epub 2018 Feb 8. PMID: 29423616.

[6] Alvarez-Perez FJ, Paiva F. Prevalence and Risk Factors for Delirium in Acute Stroke Patients. A Retrospective 5-Years Clinical Series. J Stroke Cerebrovasc Dis. 2017 Mar;26(3):567-573. doi: 10.1016/j.jstrokecerebrovasdis.2016.11.120. Epub 2016 Dec 21. PMID: 28012839.

[7] Oldenbeuving AW, de Kort PL, Jansen BP, Algra A, Kappelle LJ, Roks G. Delirium in the acute phase after stroke: incidence, risk factors, and outcome. Neurology. 2011 Mar 15;76(11):993-9. doi: 10.1212/WNL.0b013e318210411f. Epub 2011 Feb 9. PMID: 21307355.

[8] Dostovic Z, Dostovic E, Smajlovic D, Ibrahimagic OC, Avdic L, Becirovic E. PREDICTORS FOR POST- STROKE DELIRIUM OUTCOME. Mater Sociomed. 2016 Oct;28(5):382-386. doi: 10.5455/msm.2016.28.382-386. Epub 2016 Oct 17. PMID: 27999490; PMCID: PMC5149427.

[9] Miu DK, Yeung JC. Incidence of post-stroke delirium and 1-year outcome. Geriatr Gerontol Int. 2013 Jan;13(1):123-9. doi: 10.1111/j.1447-0594.2012.00871.x. Epub 2012 Jun 7. PMID: 22672215.

[10] Qu J, Chen Y, Luo G, Zhong H, Xiao W, Yin H. Delirium in the Acute Phase of Ischemic Stroke: Incidence, Risk Factors, and Effects on Functional Outcome. J Stroke Cerebrovasc Dis. 2018 Oct;27(10):2641-2647. doi: 10.1016/j.jstrokecerebrovasdis.2018.05.034. Epub 2018 Jun 29. PMID: 30172676.

[11] Dostovic Z, Smajlovic D, Ibrahimagic OC, Dostovic A. Mortality and Functional Disability of Poststroke Delirium. Mater Sociomed. 2018 Jun;30(2):95-97. doi: 10.5455/msm.2018.30.95-97. PMID: 30061796; PMCID: PMC6029905.

[12] Hosoya R, Sato Y, Ishida E, Shibamoto H, Hino S, Yokote H, Kamata T. Association between Delirium and Prehospitalization Medication in Poststroke Patients. J Stroke Cerebrovasc Dis. 2018 Jul;27(7):1914-1920. doi: 10.1016/j.jstrokecerebrovasdis.2018.02.038. Epub 2018 Mar 27. PMID: 29602617.

[13] Kozak HH, Uğuz F, Kılınç İ, Uca AU, Serhat Tokgöz O, Akpınar Z, Özer N. Delirium in patients with acute ischemic stroke admitted to the non-intensive stroke unit: Incidence and association between clinical features and inflammatory markers. Neurol Neurochir Pol. 2017 Jan-Feb;51(1):38-44. doi: 10.1016/j.pjnns.2016.10.004. Epub 2016 Oct 24. PMID: 27816188.

[14] Rice KL, Bennett MJ, Berger L, Jennings B, Eckhardt L, Fabré-LaCoste N, Houghton D, Vidal G, Gropen T, Diggs E, Barry E, St John J, Mathew S, Egger A, Ryan S, Egger R, Galarneau D, Gaines K, Ely EW. A Pilot Randomized Controlled Trial of the Feasibility of a Multicomponent Delirium Prevention Intervention Versus Usual Care in Acute Stroke. J Cardiovasc Nurs. 2017 Jan/Feb;32(1):E1-E10. doi: 10.1097/JCN.0000000000000356. PMID: 27306854.

[15] Oldenbeuving AW, de Kort PL, Kappelle LJ, van Duijn CM, Roks G. Delirium in the acute phase after stroke and the role of the apolipoprotein E gene. Am J Geriatr Psychiatry. 2013 Oct;21(10):935-7. doi: 10.1016/j.jagp.2013.01.068. Epub 2013 Aug 19. PMID: 23969090.

[16] Oldenbeuving AW, de Kort PL, van Eck van der Sluijs JF, Kappelle LJ, Roks G. An early prediction of delirium in the acute phase after stroke. J Neurol Neurosurg Psychiatry. 2014 Apr;85(4):431-4. doi: 10.1136/jnnp-2013-304920. Epub 2013 Jun 6. PMID: 23744891.

[17] Kostalova M, Bednarik J, Mitasova A, Dušek L, Michalcakova R, Kerkovsky M, Kasparek T, Jezkova M, Balabanova P, Vohanka S. Towards a predictive model for post-stroke delirium. Brain Inj. 2012;26(7-8):962-71. doi: 10.3109/02699052.2012.660510. Epub 2012 May 9. PMID: 22571183.

[18] Carin-Levy G, Mead GE, Nicol K, Rush R, van Wijck F. Delirium in acute stroke: screening tools, incidence rates and predictors: a systematic review. J Neurol. 2012 Aug;259(8):1590-9. doi: 10.1007/s00415-011-6383-4. Epub 2012 Jan 11. PMID: 22234842.

[19] Pendlebury ST, Wadling S, Silver LE, Mehta Z, Rothwell PM. Transient cognitive impairment in TIA and minor stroke. Stroke. 2011 Nov;42(11):3116-21. doi: 10.1161/STROKEAHA.111.621490. Epub 2011 Sep 8. PMID: 21903955.

[20] Klimiec E, Dziedzic T, Kowalska K, Szyper A, Pera J, Potoczek P, Slowik A, Klimkowicz-Mrowiec A. PRospective Observational POLIsh Study on post-stroke delirium (PROPOLIS): methodology of hospital-based cohort study on delirium prevalence, predictors and diagnostic tools. BMC Neurol. 2015 Jun 19;15:94. doi: 10.1186/s12883-015-0351-z. PMID: 26084453; PMCID: PMC4472262.

[21] Rollo E, Callea A, Brunetti V, Vollono C, Marotta J, Imperatori C, Frisullo G, Broccolini A, Della Marca G. Delirium in acute stroke: A prospective, cross-sectional, cohort study. Eur J Neurol. 2021 May;28(5):1590-1600. doi: 10.1111/ene.14749. Epub 2021 Feb 10. Erratum in: Eur J Neurol. 2021 Nov;28(11):3873. PMID: 33476475.

[22] Kotfis K, Bott-Olejnik M, Szylińska A, Listewnik M, Rotter I. Characteristics, Risk Factors And Outcome Of Early-Onset Delirium In Elderly Patients With First Ever Acute Ischemic Stroke - A Prospective Observational Cohort Study. Clin Interv Aging. 2019 Oct 21;14:1771-1782. doi: 10.2147/CIA.S227755. PMID: 31695347; PMCID: PMC6814355.

[23] Melkas S, Laurila JV, Vataja R, Oksala N, Jokinen H, Pohjasvaara T, Leppävuori A, Kaste M, Karhunen PJ, Erkinjuntti T. Post-stroke delirium in relation to dementia and long-term mortality. Int J Geriatr Psychiatry. 2012 Apr;27(4):401-8. doi: 10.1002/gps.2733. Epub 2011 May 10. PMID: 21560162.

[24] Dahl MH, Rønning OM, Thommessen B. Delirium in acute stroke--prevalence and risk factors. Acta Neurol Scand Suppl. 2010;(190):39-43. doi: 10.1111/j.1600-0404.2010.01374.x. PMID: 20586734.

[25] Mc Manus J, Pathansali R, Hassan H, Ouldred E, Cooper D, Stewart R, Macdonald A, Jackson S. The evaluation of delirium post-stroke. Int J Geriatr Psychiatry. 2009 Nov;24(11):1251-6. doi: 10.1002/gps.2254. PMID: 19388034.

[26] Shih HT, Huang WS, Liu CH, Tsai TC, Lu CT, Lu MK, Chen PK, Tseng CH, Jou SB, Tsai CH, Lee CC. Confusion or delirium in patients with posterior cerebral arterial infarction. Acta Neurol Taiwan. 2007 Sep;16(3):136-42. PMID: 17966952.

[27] Sheng AZ, Shen Q, Cordato D, Zhang YY, Yin Chan DK. Delirium within three days of stroke in a cohort of elderly patients. J Am Geriatr Soc. 2006 Aug;54(8):1192-8. doi: 10.1111/j.1532-5415.2006.00806.x. PMID: 16913984.

[28] Caeiro L, Ferro JM, Albuquerque R, Figueira ML. Delirium in the first days of acute stroke. J Neurol. 2004 Feb;251(2):171-8. doi: 10.1007/s00415-004-0294-6. PMID: 14991351.

[29] Hénon H, Lebert F, Durieu I, Godefroy O, Lucas C, Pasquier F, Leys D. Confusional state in stroke: relation to preexisting dementia, patient characteristics, and outcome. Stroke. 1999 Apr;30(4):773-9. doi: 10.1161/01.str.30.4.773. PMID: 10187878.

Supplemental table 2

Region*	lesion w/ delirium (n)	lesion w/out delirium (n)	Odds ratio	p-value	MNI coordinates of maximum		
					x	y	z
**Incidence**							
Right Inferior Parietal Lobule A40rv, rostroventral area 40 (PFop)	6	4	3.8	.05	36	-34	16
Right Insular Gyrus dIg, dorsal granular insula	6	2	7.9	.01	36	-12	6
Left Insular Gyrus dIg, dorsal granular insula	6	2	7.9	.01	-38	2	-2
Right Basal Ganglia dlPu, dorsolateral putamen	6	4	3.8	.05	22	0	-8
**Duration**							
	**patients w/ lesion (n)**	**PSD duration w/ lesion (days; mean, std)**	**PSD duration w/out lesion (days; mean, std)**				
Left Insular Gyrus dIg, dorsal granular insula	8	2.3 (2.1)	0.9 (2.5)	<.001	-38	2	-2
Left Basal Ganglia dCa, dorsal caudate	8	1.8 (2.4)	1.0 (2.5)	<.001	-18	-20	18

Supplemental Table 2, prognostic value of MRI lesions for delirium incidence and duration. In the 44 patients with available MRI data, delirium incidence was significantly higher when lesions occurred in either of the bilateral insular regions, right basal ganglia or right inferior parietal lobule. Odds ratios were about 4-8 times higher with lesions in the respective region. PSD duration was significantly influenced left insular or left basal ganglia lesions. Mean PSD durations were about 1-2 days longer in patients with as compared to without lesions in respective regions. Results are summarized in figure 1. Abbreviations: PSD = post-stroke delirium; w/ = with; w/out = without; MNI = Montreal Neurological Institute; std = standard deviation, * (regions were identified using the publically available brainnetome atlas)[1]

1. Fan L, Li H, Zhuo J, et al. The Human Brainnetome Atlas: A New Brain Atlas Based on Connectional Architecture. *Cereb Cortex* 2016; 26: 3508-3526. 2016/05/28. DOI: 10.1093/cercor/bhw157.

Supplemental figure 1

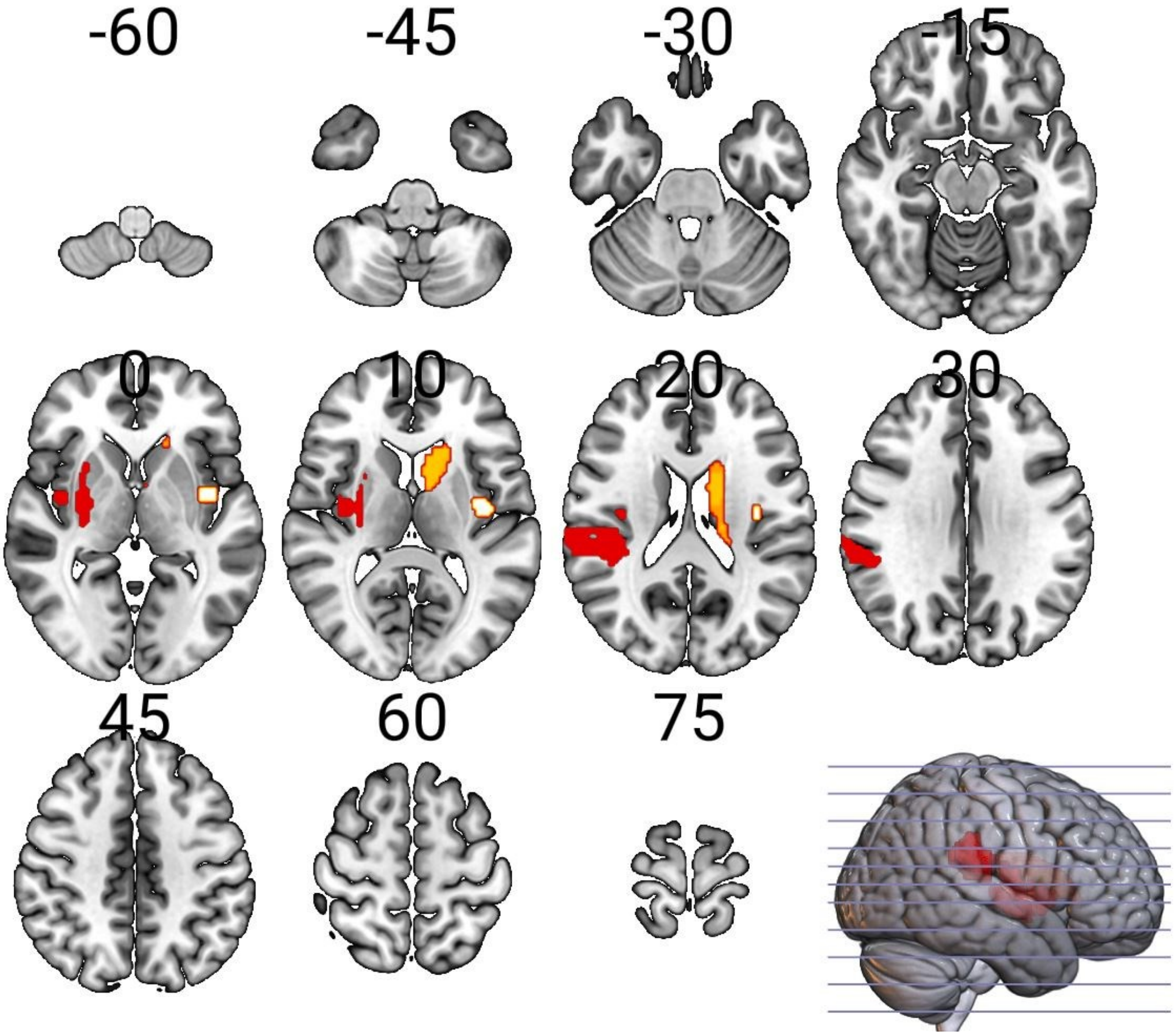

Supplemental figure 1, illustration of stroke lesion locations that were significantly associated with PSD. There were 44 patients in the study sample that received an MRI. Acute lesions were confirmed in diffusion weighted sequences, marked in the corresponding T2-weighted images and warped to a standard MRI in Montréal Neurological Institute (MNI) space (z-position of axial slices is indicated). Group data were statistically compared between patients with and without delirium. Regions highlighted in red or orange significantly predicted either PSD incidence or duration, respectively. Regions highlighted in white were significantly associated with PSD incidence and duration. Patients with PSD were more frequently affected by lesions in the basal ganglia and insular regions. Findings are furthermore summarized in supplemental table 2.
